# A *SDHB* Variant of Unknown Significance in a Patient With a Cardiac Functional Paraganglioma

**DOI:** 10.1210/jcemcr/luad093

**Published:** 2023-08-14

**Authors:** Lindsay Carafone, Adrienne Victor, Inga Harbuz-Miller

**Affiliations:** Division of Endocrinology, University of Rochester, Rochester, NY 14642, USA; Division of Hematology and Oncology, University of Rochester, Rochester, NY 14642, USA; Division of Endocrinology, University of Rochester, Rochester, NY 14642, USA

**Keywords:** cardiac paraganglioma, *SDHB*, VUS, cabozantinib

## Abstract

Cardiac paragangliomas are extremely rare tumors derived from chromaffin cells of the neural crest. Succinate dehydrogenase B (*SDHB*) mutations are associated with metastatic potential and potentially worse prognosis. Here we describe the case of a 64-year-old man who presented with chest pain, fatigue, and weight loss. Cardiac workup revealed a nearly 7-cm cardiac mass in the right lateral wall. Incisional biopsy demonstrated paraganglioma. Plasma free normetanephrine and chromogranin A were elevated. A DOTATATE positron emission tomography/computed tomography (PET/CT) revealed avidity of the mass with no evidence of distant metastases. Next-generation sequencing of the specimen demonstrated a variant of unknown significance of *SDHB* at H244D. Germline testing was negative. Surgical resection was aborted due to involvement of critical structures of the heart. Systemic treatment with the multi-tyrosine kinase inhibitor cabozantinib was initiated with subsequent improvements in biochemical markers as well as reductions in maximum standardized uptake value (SUVmax) on Ga-68 DOTATATE PET/CT. After 5 months of cabozantinib, he was unable to tolerate the side effects and external beam radiation therapy was completed. In this case, we report a novel somatic *SDHB* mutation at H244D in a sympathetic paraganglioma presenting as a cardiac mass.

## Introduction

Paragangliomas are rare extra-adrenal neuroendocrine tumors derived from chromaffin cells of the neural crest. Cardiac paragangliomas, in particular, are exceptionally rare. Both germline and somatic mutations are common in paragangliomas. This is the first known case of a missense mutation of *SDHB* at H244D.

## Case Presentation

A 64-year-old man presented with 6 months of intermittent chest pain, palpitations, progressive fatigue, presyncopal episodes, and a 15-pound unintentional weight loss. His past medical history was notable for type 2 diabetes mellitus, hypertension, and hyperlipidemia. Family history was notable for coronary artery disease in his father who underwent coronary artery bypass graft at age 70 years. There was no family history of pheochromocytoma, paraganglioma, or other endocrine disorder.

## Diagnostic Assessment

**Figure 1.**
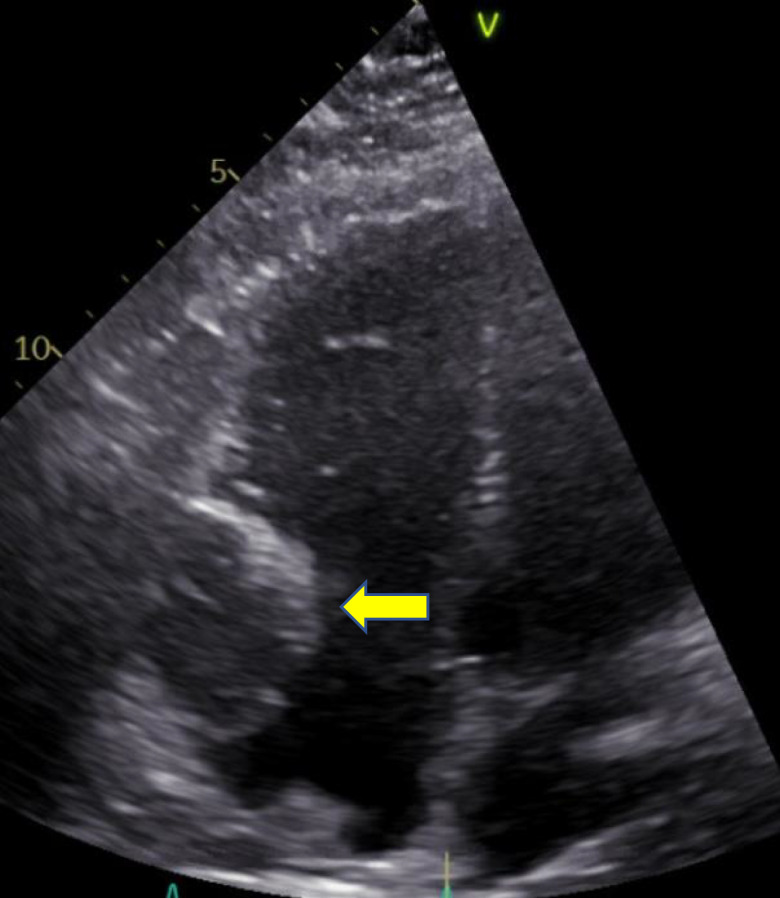
Echocardiogram: large heterogenous mass in the right atrium appearing adherent to the lateral wall.

**Figure 2.**
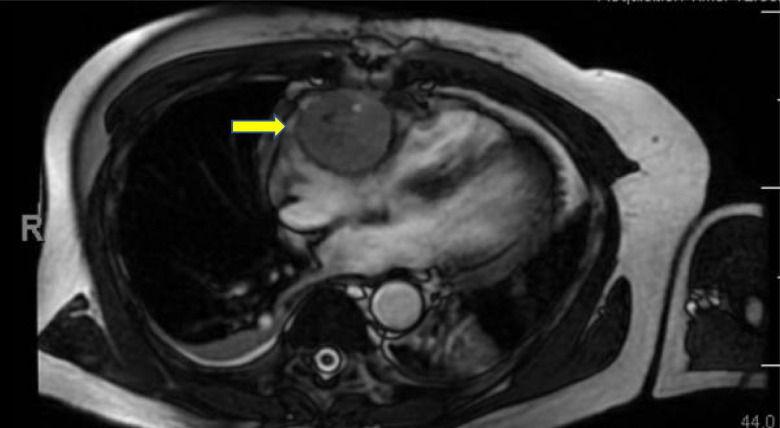
Cardiac MRI: 6.9 × 5.8 × 4.9 cm mass involving the right lateral wall, atrioventricular groove, and encasing the right coronary artery.

**Figure 3.**
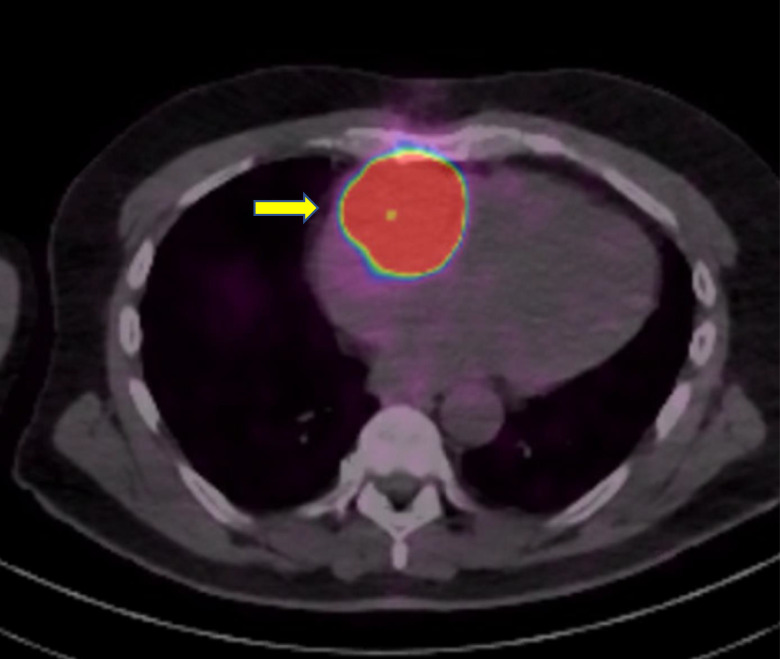
Initial DOTATATE PET/CT: DOTATATE avidity within the region of the right-sided cardiac mass. SUVmax 94.

Nuclear stress test showed no evidence of ischemia. Echocardiography revealed a large right-sided cardiac mass appearing adherent to the right lateral wall, trace tricuspid regurgitation, normal left ventricular ejection fraction, and no evidence of diastolic dysfunction (Fig. 1). The mass was further characterized with cardiac magnetic resonance imaging revealing a 6.9 × 5.8 × 4.9 cm mass in the lateral right atrial wall and right atrioventricular groove, encasing the right coronary artery (Fig. 2). Incisional biopsy was performed, and pathology revealed nests and packets of round and multinucleated cells with some nuclear atypia and abundant cytoplasm. Tumor cells were strongly positive for chromogranin, synaptophysin, GATA3, and S100, consistent with paraganglioma. The tumor cells were negative for pancytokeratin and CD10. Laboratory tests revealed plasma free normetanephrine of 1.87 nmol/L (0.068 mcg/dL) [normal range, 0-0.89 nmol/L], plasma free metanephrine 0.14 nmol/L (0.005 mcg/dL) [normal range, 0-0.49 nmol/L], and chromogranin A 1038 ng/mL (103.8 mcg/dL) [normal range, <93 ng/mL]. An iodine-123 meta-iodobenzylguanidine (MIBG) scan revealed persistently elevated MIBG activity in the region of the cardiac mass. Ga-68 DOTATATE positron emission tomography/computed tomography (PET/CT) revealed a DOTATATE avid cardiac mass and no other avid areas to suggest distant metastases (Fig. 3).

Next-generation sequencing was performed on the tissue biopsy. This showed a *SDHB* mutation c.730C > G (p.H244D), which is a variant of unknown significance (VUS) with a variant allele frequency (VAF) of 62.2%. VAF represents the percentage of sequence reads with the variant divided by the total number of reads at that locus. In other words, VAF represents the percentage of DNA molecules in the specimen that have the variant. If the VAF is approximately 50% or 100%, this variant may represent a germline mutation with heterozygous or homozygous loci, respectively [1]. VAF in somatic testing can vary depending on tumor heterogeneity and contamination from normal cells [2]. In this patient's case, subsequent germline testing was performed with an 84-gene panel using Invitae and was negative for a *SDHB* variant. The patient's variant therefore represents a somatic mutation.

## Treatment

Surgical resection is the first-line treatment for cardiac paraganglioma and was therefore attempted in this patient. A sternotomy was performed and upon opening the pericardium, the mass was found to involve the right ventricle and was exquisitely vascular. Further evaluation demonstrated involvement of the tricuspid valve, which was not appreciated on any preoperative imaging. Ultimately, it was deemed unsafe to resect the mass due to the involvement of critical cardiac structures, and surgical resection was aborted.

As the paraganglioma was unresectable, systemic therapy was instead pursued with cabozantinib 60 mg daily.

## Outcome and Follow-up

The patient required dose reduction of cabozantinib to 40 mg daily because of palmar-plantar erythrodysesthesia, which is a known adverse effect of cabozantinib. At 2-month follow-up assessment, repeat Ga-68 DOTATATE PET/CT showed increased central photopenia with 54 standardized uptake value (SUV)max from previously 94 SUVmax. Biochemical assessment revealed chromogranin A 519 ng/mL (51.9 mcg/dL) and plasma free norepinephrine 1.43 nmol/L (0.052 mcg/dL), which corresponded to 50.0% and 23.5% reductions, respectively. Plasma free metanephrine was 0.23 nmol/L (0.008 mcg/dL), which remained within the normal range.

Cabozantinib was stopped approximately 5 months after initiation due to inability to tolerate side effects of nausea, diarrhea, and palmar-plantar erythrodysesthesia. Chromogranin A subsequently increased to 1122 ng/mL (112.2 mcg/dL) and plasma free norepinephrine increased to 2.35 nmol/L (0.085 mcg/dL). DOTATATE PET/CT showed increased SUVmax of 165. He was then treated with external beam radiation (EBRT) in 25 fractions for a total dose of approximately 45 Gy. Following EBRT, chromogranin A decreased by 24% to 850 ng/mL (85 mcg/dL), plasma free norepinephrine decreased by 38% to 1.45 nmol/L (0.053 mcg/dL), plasma free metanephrine remained normal at 0.11 nmol/L (0.004 mcg/dL), and SUVmax decreased to 80 on DOTATATE PET/CT.

## Discussion

Cardiac paragangliomas represent a small fraction of these rare endocrine neoplasms. Guidelines recommend that all paragangliomas be screened for mutations in *SDHx* genes. *SDHB* mutations are associated with potentially worse prognosis [3] and higher rates of metastatic disease [4]. Here we report the first case of a somatic mutation of *SDHB* at H244D in an unresectable cardiac paraganglioma treated with cabozantinib and EBRT. H244D is a missense mutation that replaces histidine with aspartic acid and thereby changes the side chain from positively to negatively charged.

When a mutation is known to be associated with a disease, it is classified as a pathogenic variant. The mutation in this case represents a VUS, indicating that there is currently insufficient evidence to determine its role in disease. For any given VUS, the suspicion for pathogenicity can range from low to high. Given our knowledge of the pathogenic role of *SDHB* mutations in paragangliomas, our suspicion for pathogenicity is high for this patient's VUS. The classification criteria by the American College of Medical Genetics and Genomics–Association for Molecular Pathology (ACMG-AMP) are stringent and result in a relatively large number of variants falling under the VUS category in order to reduce variants being classified as pathogenic without enough supporting evidence [5]. Different approaches can be used to aid in the determination of pathogenicity, including cumulative evidence from other patients with the same phenotype and VUS as well as in vivo or in vitro studies to observe changes in phenotype with the mutation [6]. We suspect that in the future as further evidence accumulates from other patients with this mutation, that it may be reclassified as “likely pathogenic” (greater than 90% certainty of the variant being disease-causing) or “pathogenic” per ACMG-AMP guidelines for interpretation of sequence variants. The patient's c.730C > G (p.H244D) variant of *SDHB* has not been previously reported; therefore, it is expected that it is exceedingly rare or not present in the general population. A histidine to tyrosine substitution at position 244 has been reported once in the Exome Aggregation Consortium (ExAC) database with an allele frequency of 3.98e-6.

Cardiac paragangliomas are associated with mutations in *SDHB*, *SDHD*, and *SDHC*. In a retrospective study of 13 patients with cardiac paragangliomas who underwent genetic testing, 77% of patients had an *SDHx* mutation (3 *SDHB*, 4 *SDHD*, and 3 *SDHC*) [7]. There are few published cases describing the clinical course and outcomes of cardiac paragangliomas with *SDHB* mutations. Out of 11 cases in the literature describing cardiac paragangliomas with *SDHB* mutations, 10 patients underwent surgical resection. Details regarding follow-up and long-term outcomes were not available in the majority of cases, so it is unknown whether these paragangliomas recurred following surgical resection, and what, if any, systemic therapies were subsequently used. In only 1 of the 11 cases, surgery was deferred due to location. That patient underwent alcohol ablation and coiling of a branch of the right coronary artery which supplied the tumor; he subsequently received palliative chemotherapy [8].

Surgical resection is the first-line treatment of cardiac paragangliomas. In this patient's case, surgical resection was aborted due to risk of injury to critical cardiac structures. When a paraganglioma is unresectable or surgery poses unacceptable risks, systemic therapy can be used. There are several options for systemic therapy in the treatment of paragangliomas; however, the evidence is largely based on small, retrospective studies. Tumors with *SDHB* mutations tend to have abnormal angiogenesis with hypervascularity. Cabozantinib is a multi-tyrosine kinase inhibitor and potent antiangiogenic agent that can be used when paragangliomas cannot be treated surgically or are metastatic. A phase 2 clinical trial (NCT02302833) is currently recruiting patients to study cabozantinib in pheochromocytomas and paragangliomas with locally advanced or metastatic disease not amenable to surgery. Preliminary data demonstrated an objective response rate of 40% and clinical benefit rate of 90% [9]. The patient in our case had improvement in biochemical markers and reduction in DOTATATE avidity with cabozantinib, but unfortunately treatment was discontinued due to intolerance of adverse effects.

This case also demonstrated reduction in biochemical markers and DOTATATE avidity with EBRT with a dose of 45 Gy, following failure to tolerate ongoing systemic therapy. Radiation therapy is not well studied in cardiac paraganglioma. However, radiation has been used in the treatment of paragangliomas, and prior data suggests that doses exceeding 40 Gy yield greater local control than lower doses. The local control resulting from EBRT appears to be due to stabilization of disease as opposed to a substantial reduction of tumor volume [10].

It is too early to know the clinical relevance of the novel *SDHB* mutation in this case; however, it may prove clinically meaningful in characterizing cardiac paraganglioma. Radionuclide therapy with Iobenguane 131, which utilizes the noradrenaline transporter present on paragangliomas, has also shown promise in treatment of pheochromocytomas and paragangliomas, and depending on the patient's course may be a next step in his treatment approach. “Cold” somatostatin analogs (octreotide LAR and lanreotide) have also been used in locally unresectable or metastatic paragangliomas. Alternative potential treatment options in cases of unresectable paraganglioma include chemotherapy with cyclophosphamide, vincristine, and dacarbazine (CVD), as well as temozolomide (particularly in *SDHx-*mutated tumors). Finally, peptide receptor radionuclide therapy (PRRT), such as ^177^Lu-DOTATATE, could be considered for this patient given the DOTATATE avidity of the tumor [4].

## Learning Points

- All paragangliomas should be screened for SDHx mutations.
- SDHB mutations in paragangliomas are associated with higher metastatic potential and potentially worse prognosis.
- While surgical resection is the first-line treatment for cardiac paragangliomas, this is not always possible due to risk of injury to critical structures of the heart. In these cases, systemic therapy can be pursued.
- Cabozantinib is a multi-tyrosine kinase inhibitor and potent antiangiogenic agent that can be considered for treatment of unresectable or metastatic paragangliomas.
- Radiation therapy may be useful in local control of unresectable cardiac paragangliomas.

## Contributors

All authors made individual contributions to authorship. L.C. was involved in manuscript preparation and submission. I.H.M. and A.V. were involved in the diagnosis and management of this patient, and manuscript submission. All authors reviewed and approved the final draft.

## Data Availability

Data sharing is not applicable to this article as no datasets were generated or analyzed during the current study.

## Abbreviations

ACMG-AMP: American College of Medical Genetics and Genomics–Association for Molecular Pathology
EBRT: external beam radiation
MIBG: meta-iodobenzylguanidine
PET/CT: positron emission tomography/computed tomography
SUVmax: maximum standardized uptake value
VAF: variant allele frequency
VUS: variant of unknown significance
